# A novel method for assessment of human midpalatal sutures using CBCT-based geometric morphometrics and complexity scores

**DOI:** 10.1007/s00784-023-05055-6

**Published:** 2023-05-13

**Authors:** Stratos Vassis, Oskar Bauss, Beatrice Noeldeke, Mohammedreza Sefidroodi, Peter Stoustrup

**Affiliations:** 1 Section of Orthodontics, Department of Dentistry and Oral Health, Aarhus University, Vennelyst Blvd. 9, 8000 Aarhus, Denmark; 2 Orthodontic Practice, Luisenstrasse 10/11, 30159 Hannover, Germany; 3 Leibniz University Hannover, Hannover, Germany

**Keywords:** Suture morphology, Suture complexity, Midpalatal suture, CBCT, Complexity score

## Abstract

**Introduction:**

Management of dentofacial deficiencies requires knowledge about sutural morphology and complexity. The present study assesses midpalatal sutural morphology based on human cone-beam computed tomography (CBCT) using geometric morphometrics (GMM) and complexity scores. The study is the first to apply a sutural complexity score to human CBCT datasets and demonstrates the potential such a score has to improve objectiveness and comparability when analysing the midpalatal suture.

**Materials and methods:**

CBCTs of various age and sex groups were analysed retrospectively (*n* = *48*). For the geometric morphometric analysis, landmark acquisition and generalised Procrustes superimposition were combined with principal component analysis to detect variability in sutural shape patterns. For complexity analysis, a windowed short-time Fourier transform with a power spectrum density (PSD) calculation was applied to resampled superimposed semi-landmarks.

**Results:**

According to the GMM, younger patients exhibited comparable sutural patterns. With increasing age, the shape variation increased among the samples. The principal components did not sufficiently capture complexity patterns, so an additional methodology was applied to assess characteristics such as sutural interdigitation. According to the complexity analysis, the average PSD complexity score was 1.465 (standard deviation = 0.010). Suture complexity increased with patient age (*p* < 0.0001), but was not influenced by sex (*p* = 0.588). The intra-class correlation coefficient exceeded 0.9, indicating intra-rater reliability.

**Conclusion:**

Our study demonstrated that GMM applied to human CBCTs can reveal shape variations and allow the comparison of sutural morphologies across samples. We demonstrate that complexity scores can be applied to study human sutures captured in CBCTs and complement GMM for a comprehensive sutural analysis.

## Background

Craniofacial sutures act as active growth sites, absorb mechanical stresses and protect the brain [1]. These functions impact the overall skull shape and also sutural morphology and complexity [2, 3]. In case of abnormal craniofacial development, sutures may be surgically or orthopaedically distracted to manipulate growth and treat dentofacial deficiencies [4]. Thus, the maxillary midpalatal suture plays an important role in maxillary development and growth. Furthermore, the outcome of treating skeletal maxillary malformations depends on the morphology and complexity of this suture [5–7]. Midpalatal suture morphology may vary considerably between sexes and across developmental stages [8]. This variability complicates treatment of skeletal maxillary malformations [9]. More specifically, findings suggest that pronounced sutural interdigitation hinders transverse expansion success [10]. Hence, analysis of the patient’s sutural characteristics provides insights into the midpalatal suture’s morphology and degree of interdigitation and may support medical decision-making and enhance treatment success [5].

Previous work assessing midpalatal sutural morphology in humans has mainly focused on histological and radiographic observations. Histological research has described the midpalatal suture as a butt joint [7, 11, 12], which evolves into other, more complex, highly interdigitated joint types during ontogeny and in reaction to mechanical stress [1, 3, 13–15]. Histologic and micro-radiographic frontal sections have shown the suture as undulating in the juvenile period, whereas it assumes a sinuous course with increasing interdigitation in the adolescent period [8]. Histological research has shown that midpalatal suture morphology develops highly interdigitated patterns over time [8, 9, 16] and that morphology varies considerably between age groups [5, 16, 17]. In recent years, radiographic approaches have used cone-beam computed tomography (CBCT) to visualise palatal suture morphology. More specifically, Angelieri et al. morphologically classified midpalatal sutures into stages from A to E as observed on CBCT [7, 16, 18]. Another study detected variations in sutural morphology using flat-panel volume computed tomography of animals [11]. However, no quantitative analysis based on objective metrics has yet been presented in the literature. Consequently, the comparability of sutural shapes can be compromised, and more studies are needed to determine how CBCT can be used to describe sutural features [19].

To improve the objectiveness and comparability and to account for several morphological sutural features simultaneously, geometric morphometrics (GMM) and complexity scores may be implemented to evaluate suture morphology and complexity, respectively. GMM is an approach to statistically evaluate shapes based on landmark coordinates. Firstly, to make shapes comparable, GMM removes size, rotation and translation from the landmark’s configurations. Secondly, principal component analysis (PCA) is performed. As a statistical method, PCA extracts relevant information from datasets containing various variables and summarises this information in a new, smaller set of variables: the principal components. Thereby, the dimensionality of the data is reduced, and the overall variability of shapes becomes detectable [20, 21]. Focusing on complexity as one aspect of morphology, complexity scores mathematically integrate interdigitation characteristics such as amplitude, number of interdigitation and looping patterns into a single score [3]. Among available complexity scores, the windowed short-time Fourier transform (STFT) with a power spectrum density (PSD) calculation appears most promising to comprehensively capture sutural complexity. However, the PSD complexity score has been calculated only for sutures of diverse mammalian taxa on X-ray microtomography [3] and has not been applied to CBCT of human midpalatal sutures.

This study aimed to demonstrate that the combination of GMM and sutural complexity scores may constitute a novel and comprehensive CBCT-based sutural analysis in humans. We retrospectively analysed midpalatal sutures on CBCT by applying GMM. This study was the first to calculate a sutural complexity score based on human CBCT.

## Methods

### Data collection

This retrospective study was based on CBCTs from a sample of consecutive patients treated in a German orthodontic and maxillofacial surgery clinic between January 2020 and July 2022. For the included patients, maxillary CBCT examinations were performed based on one the following indications: position of severely impacted teeth, bone dimensions prior to implant placement or implant site dimensions. Only patients with already existing CBCTs were included in this study. Because of the retrospective design of the study, no ethical approval was required. However, every patient gave written consent for the use of their medical data for scientific purposes related to this specific study. The data files were processed anonymously.

The exclusion criteria were previous rapid maxillary expansion, cleft lip and palate, and impaired bone metabolism due to medication or artefacts in the same plane as the midpalatal suture (e.g. the transpalatal arch). In addition, patients with already fused midpalatal sutures (stage E suture fusion according to Angelieri) [7] were excluded because their midpalatal suture outline cannot be traced and their parasutural bone density is the same as that of other palatal regions [16]. Notably, the midpalatal suture may remain open throughout life [5], which allowed us to include patients with higher age in the sample.

To avoid interobserver bias, one author conducted the data analysis.

### Method

#### Cone-beam computed tomography

The CBCTs were generated using Orthophos® XG 3D (Dentsply Sirona, Bensheim, Germany). The radiation dose ranged from 91 mGy*cm^2^ to 781 mGy*cm^2^. Volume sizes varied from 5 × 5.5 cm through 8 × 8 cm to 11 × 10 cm. The acquisition time was 4300 ms for 42 patients and 2500 ms for six patients.

#### Data preparation

**Fig. 1:**
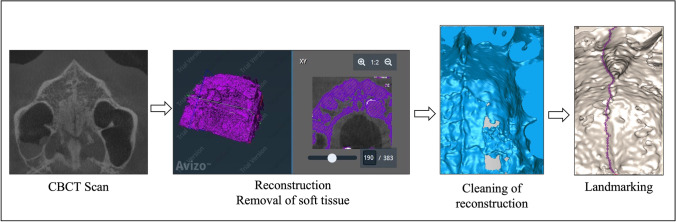
Process of data preparation and analysis. Note: The figure shows, from left to right, CBCTs viewed in RadiAnt DICOM Viewer, segmentation of bony structures in Avizo, processing of three-dimensional surfaces in Geomagic and landmarking of two-dimensional photographs using R packages. Abbreviations: CBCT, cone-beam computed tomography

Data preparation comprised several steps, as depicted in Fig. 1. First, the CBCTs were converted into anonymised DICOM files and exported using RadiAnt DICOM Viewer [22]. Second, the exported files were reconstructed in Avizo v.9.3 software (FEI, Hillsboro, OR, USA) and Geomagic Wrap (3D Systems) so that only the cranial segments were displayed in the three-dimensional isosurface, based on case-specific thresholds to optimise sutural traceability. Third, the midpalatal suture was photographed using uniform positioning to control the degree of parallax in the R package ‘rgl’ [23]. To check for robustness, the data were additionally prepared using the open-source software 3DSlicer [24] as an alternative to Avizo and Geomagic. These steps produced two-dimensional digital photographs of the midpalatal suture, which were used for the subsequent analysis.

#### Geometric morphometric methods

GMM can capture morphological structure, overall phenotype and three-dimensional profiles of sutures without information loss during shape analysis [19, 25, 26]. For the GMM, we followed White et al.: first, we manually positioned two-dimensional landmarks on the digital photographs. Based on a sliding algorithm to consistently place 500 semi-landmarks per suture, we resampled them the semi-landmarks using the R package ‘Stereomorph’ to avoid loss of morphological complexity [27, 28]. Furthermore, to reduce differences between individuals and align the positions of their landmarks, generalised Procrustes superimposition was performed. Procrustes superimposition transforms raw landmarks into shape coordinates by centring (translating), resizing and rotating the landmarks. Thereby, the landmarks are converted into so-called semi-landmarks [19, 29–32]. The superimposed semi-landmarks were then analysed using PCA. In this study, PCA was implemented to identify the main components of shape variation across the sample. As a result, the samples can be mapped into a common coordinate system, the morphospace, where sutural morphology can be analysed individually or compared among individuals [33–36].

#### Complexity analysis

For the complexity analysis, we implemented the windowed short-time Fourier transform with a power spectrum density calculation to the Procrustes superimposed semi-landmarks. The PSD complexity score is well suited to capture the characteristics of interdigitations, loop patterns and amplitude [3]. A score was computed for each suture by averaging the squared windowed short-time Fourier transform coefficients over each frequency across the local transforms and summing the averages at each harmonic, following the method proposed by Allen et al. [3, 37]. Low PSD complexity scores suggest a straight outline, whereas increasing values indicate a progression towards interdigitated sutural outlines with pronounced loops and amplitudes [15]. We computed the complexity score using the R packages ‘e1071’ and ‘stft’ [23]. The analysis was based on White et al., where further details on the methodology and codes for the implementation in R are provided [38, 39].

#### Statistical analysis

A Shapiro–Wilk test suggested that the data were normally distributed, so the complexity scores were analysed using analysis of variance (ANOVA) to determine differences among the age quartiles and sex groups. For detecting differences among the specific age groups, pairwise comparisons based on the *t* tests with Holm-Bonferroni [40] corrections were conducted. Differences were considered significant if the corresponding *p* values were lower than 0.05. Intra-rater reliability was evaluated for the landmark placement in R as the same author acquired all the landmarks again after a period of one month. Reliability was indicated by the intra-class correlation coefficient (ICC).

## Results

The applied exclusion criteria produced a final sample size of 48 patients. The sample was divided by the patient’s age, yielding in four groups comprising the four quartiles, as shown in Table 1.

**Table 1:** Sample characteristics

Group	Age range (in years)	Sex		
		Male	Female	Total
1	≤ 14	5	7	12
2	> 14 to ≤ 21	6	6	12
3	> 21 to ≤ 49	5	7	12
4	> 49	4	8	12
Total		20	28	48

### Geometric morphometrics: principal component analysis

**Fig. 2:**
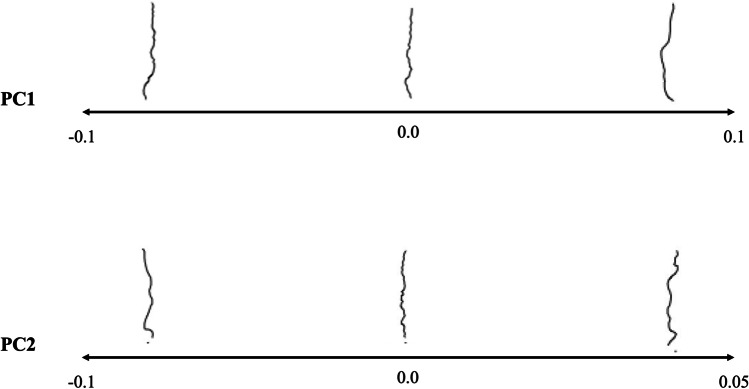
GMM results: suture morphologies along PC1 and PC2. Note: Patients with low PC1 values showed a convex suture outline, whereas patients with high PC1 values recorded a concave outline. Sutures located on the low end of the PC2 axis exhibited a loop to the left in the posterior part of the suture, which was not the case for samples with high PC2 values. Abbreviations: GMM, geometric morphometrics; PC, principal component

The PCA of the Procrustes superimposed two-dimensional semi-landmarks revealed that 13 principal components (PC) were sufficient to summarise more than 95% of the total morphological variance of the midpalatal suture. PC1 accounted for 39% of the variance; PC2 for 18%. As visualised in Fig. 2, PC1 was associated with trends related to the overall suture outline: lower values were associated with a convex form, whereas the higher PC1 values indicated a concave sutural outline. Lower PC2 values seemed to indicate a major loop to the left in the posterior part of the suture, whereas samples with higher PC2 values did not exhibit this loop in the posterior part. The morphological analysis detected no clear patterns regarding number of interdigitations related to PC1 or PC2.

**Fig. 3:**
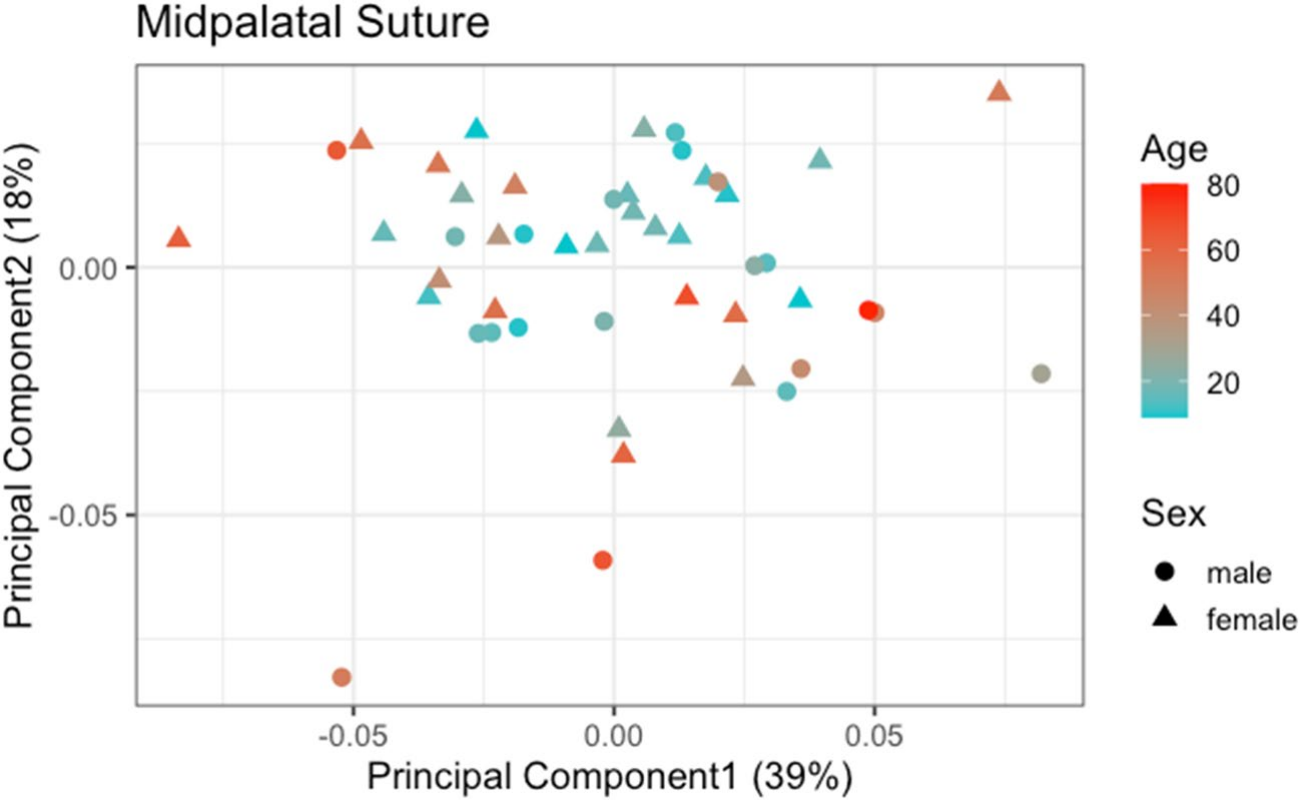
Sampling distribution along principal component 1 and principal component 2, divided by age and sex

Mapping patients into morphospace along PC1 and PC2 showed that individuals with comparatively lower age tended to be clustered in the centre of the morphospace, as shown in Fig. 3. In contrast, older individuals were more likely to be located in the periphery of the morphospace. The relatively younger patients exhibited largely similar sutural morphological characteristics as expressed by the two main principal components. In contrast, older patients displayed a higher degree of variation regarding their sutural morphology. Hence, these results suggest a higher morphological variation along PC1 and PC2 with increasing age. Regarding the patient’s sex, no clear pattern was observed.

### Power spectrum density complexity scores

To determine sutural complexity, a PSD complexity score was computed for each sample, with higher values indicating higher complexity. The complexity analysis revealed an average PSD score of 1.465 with a standard deviation of 0.010 across the whole sample. For males, the average PSD complexity score was 1.466, whereas it was 1.464 for females. With progressing age, the PSD complexity score increased to values of 1.459 (group 1), 1.460 (group 2), 1.466 (group 3), and 1.474 (group 4), indicating increasing complexity.

**Fig. 4:**
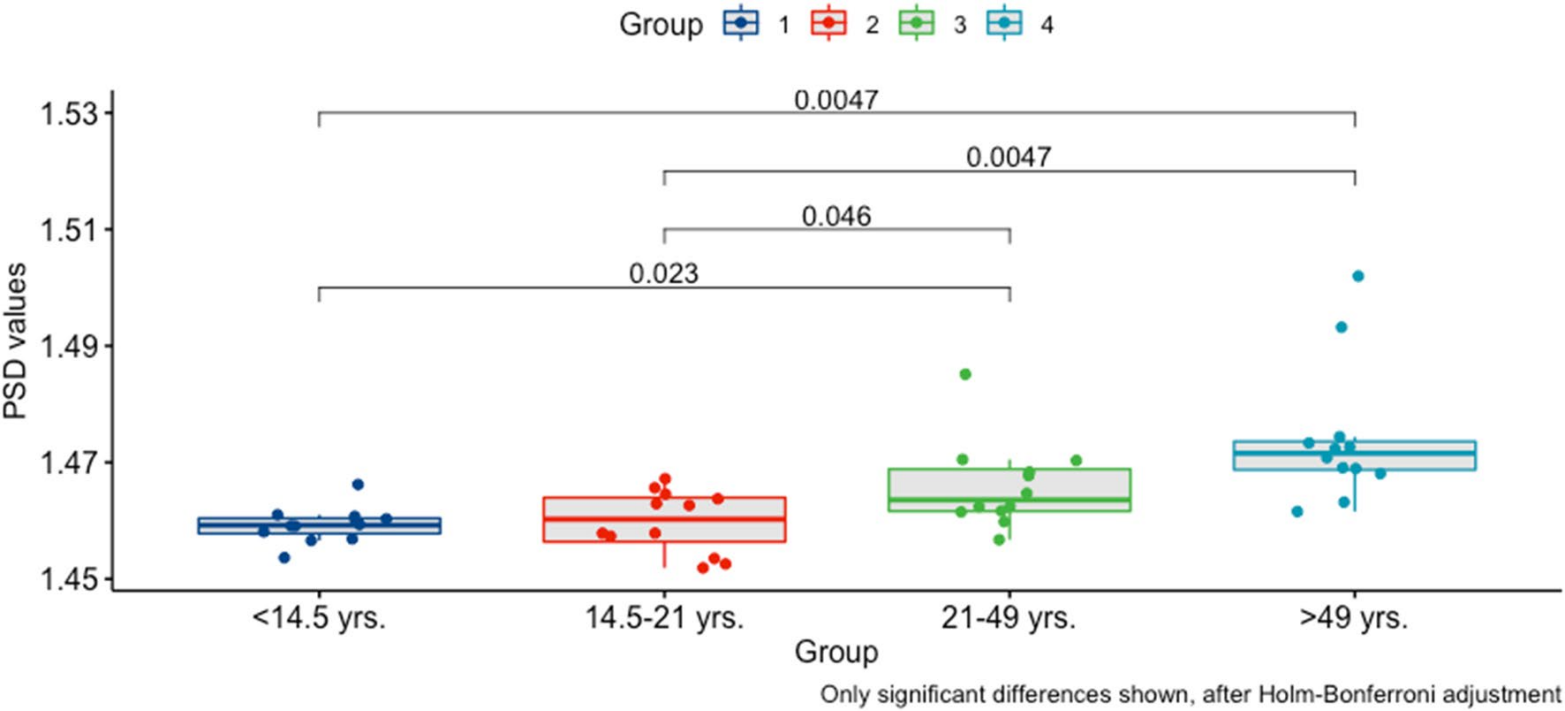
Post hoc pairwise *t* tests comparing PSD complexity scores among age groups. Abbreviation: PSD, power spectrum density

The ANOVA demonstrated a significant effect of age on midpalatal suture complexity (*p* < 0.0001, degrees of freedom (DF) = 1, *F* = 21.346). In contrast, the variable sex did not significantly impact the complexity score (*p* = 0.588, DF = 1, *F* = 0.298). Furthermore, the interaction of age and sex did not significantly influence complexity (*p* = 0.848, DF = 1, *F* = 0.037). An intergroup *t* test to assess differences among the age groups demonstrated differences that were significant at the 5% level among age group 1 and 4 (*p* = 0.001), 1 and 3 (*p* = 0.014), 2 and 3 (*p* = 0.030) and 2 and 4 (*p* = 0.002). After Holm-Bonferroni correction, the same differences remained significant, as shown in Fig. 4.

The results remained robust when outliers were excluded. The ICC was above 0.9, indicating a high intra-rater reliability. To assess the impact of the software used, the whole analysis, from reconstruction to statistical analysis, was repeated in open-source software (3D Slicer) as an alternative to segmentation and reconstruction in Avizo and Geomagic. The results remained robust to software changes.

## Discussion

This study is the first to apply a sutural complexity score to human CBCTs. More specifically, it combined GMM with a complexity score to analyse midpalatal sutures, a surface of orthodontic interest. As GMM is based on statistical calculations and the complexity score provides a single value for each sample, this approach contributes to improving the interpretability, objectiveness, and comparability of suture analysis.

The methodology proposed in this study is relevant for clinicians and researchers. For clinicians, the analysis can contribute to supporting treatment planning with respect to surgical or non-surgical corrections of maxillary transverse discrepancies [5]. As age constitutes one of several influencing factors, a complexity score that can assess sutural complexity for each patient individually offers an additional indicator for predicting the success of sutural distractions. For researchers, the proposed analysis provides a way forward towards robust and comprehensive shape analysis for CBCTs of humans that may also be used for analysis of other shapes of interest.

GMM is advantageous compared with linear measurements for analysing sutural shapes because linear measurements can be biased due to an arbitrary focus on certain sutural parts, while other sections are neglected, resulting in an incomplete shape analysis [19]. In contrast, GMM considers the shape as a whole and removes the effects of scale, translation and rotation. As a result, the pure shapes, which are mapped onto the same coordinate system using landmarks, are comparable, and any differences between sutures are truly caused by differences in their shapes. Challenges related to GMM include the landmark number, placement criteria and landmark homology between samples [19]. We addressed these challenges by resampling 500 semi-landmarks for each suture and sliding them along the curve to match their positions with the reference configuration based on the principle of minimising the Procrustes distance [41]. Another limitation relates to the interpretation of the principal components because patterns might not be uniquely identifiable and their identification might require clinical experience [19]. Lastly, in our study, neither PC1 nor PC2 seemed to capture progression of the sutural interdigitations. This suggests that another method, such as the PSD complexity score, is required to capture such characteristics as a supplement to GMM.

By quantifying midpalatal suture complexity in a complexity score, this study expanded the limited research on the complexity of this suture and showed that if it is based on a windowed STFT with PSD, the complexity score can be applied to CBCT datasets of human sutures. Our result demonstrated that sutural complexity increased with higher patient age, which is in line with histological, histomorphometric and radiological research approaches, all of which have demonstrated that the midpalatal suture develops highly interdigitated patterns over time [8, 9, 16]. Advancing previous approaches, the PSD complexity score expresses several characteristics such as amplitude, number of interdigitation and looping patterns in one score [3]. According to a recent study comparing all available complexity scores in mammals, the STFT with PSD calculation captured the above-mentioned characteristics better than other available scores: owing to its Fourier foundation, it was robust as the statistical transformations captured discrete and non-stationary characteristics [3]. The PSD complexity score is of orthodontic interest because it can support the categorisation of patients with respect to suture complexity and therefore inform treatment planning. However, calculating the complexity score involves several steps of data preparation and analysis. This requires knowledge of and access to the relevant software. Nevertheless, this study indicates a high potential of calculating PSD complexity scores for suture evaluation and comparisons.

The conducted analysis investigated age and sex as determinants of sutural morphology. Our results indicated that age significantly increased complexity, which contradicts the results of Korbmacher et al., who conclude that interdigitation is not age-dependent [42]. Yet, Korbmacher et al. calculated complexity based on linear measurements using the sutural interdigitation index [42]. Although such a sutural interidigitation index captures the number of interdigitations well, the PSD score applied in our study additionally captures interdigitation amplitude [3]. More precisely, the PSD score differentiates between shallower lobes and fewer deeper lobes, in contrast to the linear length measurements [3, 37]. These differences in the results based on different complexity metrics suggest that age might increase sutural complexity due to increased interdigitation amplitudes.

Other factors that were not considered in this study may possibly impact suture morphology, too. For example, Cheronet et al. concluded that structural factors such as the position along the cranial vault and adjacent sutures play an essential role for midpalatal suture morphology. Another structural factor not considered in our study, but related to rapid maxillary expansion success is the age-progressive bone obliteration of the midpalatal, pterygopalatinal and pterygomaxillary suture [43]. Also, extrinsic parameters such as mechanical forces or genetic factors may possibly have an impact [44]. Another limitation of our study relates to the mean sample age; due to greater radiation risk for young children our access to data for these age groups was limited. In our study, the youngest age group ranged from 9 to 14 years. In this context, Kinzinger et al. detect structural changes affecting the outcome of the rapid maxillary expansion, occurring after 10 to 12 years of age [45].

Also, it is unclear to which extent the CBCT resolution affects the complexity score due to compromised traceability of the suture outline. To improve suture outline traceability, we set case-specific thresholds, which is associated with a risk of receiving different palate shapes.

Future research should further investigate the potential and clinical relevance of complexity scores based on human CBCTs using larger sample sizes. Research could focus on whether the score can support decision-making in orthodontic treatment, such as the decision to surgically or non-surgically correct maxillary transverse discrepancies. Future work could analyse morphological shape variations, reveal shape patterns and calculate complexity scores of patients with craniosynostosis based on the methodologies applied in this paper.

## Conclusions

This study performed a geometric morphometric analysis and a complexity analysis of human midpalatal sutures. Applied to CBCTs, the methodologies revealed shape variation and quantified complexity. Thereby, our study proves the applicability of complexity scores to human CBCTs of palatal sutures, contributing to comprehensive sutural assessments.

## Data Availability

For data sensitivity reasons, the dataset was not shared or made available online.

## Abbreviations

ANOVA: Analysis of variance
CBCT: Cone-beam computed tomography
DF: Degree of freedom
GMM: Geometric morphometrics
ICC: Intra-class correlation coefficient
PC: Principal component
PCA: Principal component analysis
PSD: Power spectrum density
STFT: Short-time Fourier transform
